# Case report: Tocilizumab for hypersensitivity reaction after oxaliplatin in a patient with NK/T-cell lymphoma

**DOI:** 10.3389/fphar.2024.1471038

**Published:** 2025-01-15

**Authors:** Juanyan Liao, Ming Jiang

**Affiliations:** ^1^ Department of Biotherapy, Cancer Center, West China Hospital, Sichuan University, Chengdu, Sichuan, China; ^2^ Department of Head and Neck Oncology, Cancer Center, West China Hospital, Sichuan University, Chengdu, Sichuan, China

**Keywords:** tocilizumab, hypersensitivity reaction, oxaliplatin, NK/T-cell lymphoma, IL-6

## Abstract

Oxaliplatin-induced hypersensitivity reactions (HSRs) are commonly encountered in first-line therapies for various malignancies. Recent research indicates that these reactions can include cytokine release reactions (CRRs), which are characterized by a marked increase in interleukin-6 (IL-6) levels, sometimes rising as much as 40-fold. Standard management strategies for HSRs typically involve desensitization protocols and routine treatments. However, these conventional approaches may be insufficient for managing CRRs. Preliminary studies suggest that tocilizumab, an IL-6 receptor (IL-6R) antagonist, may play a crucial role in mitigating CRRs. In our case, a 65-year-old male with stage IV extranodal NK/T-cell lymphoma developed a severe HSR on day 1 following the infusion of oxaliplatin during his fourth chemotherapy cycle. This reaction was marked by a substantial increase in IL-6 levels. Despite the administration of standard treatments, including epinephrine and corticosteroids, the patient required ventilatory support and vasopressors on day 1. On day 2, tocilizumab was administered, resulting in a rapid and significant reduction in IL-6 levels. Subsequently, the patient’s symptoms, including fever, dyspnea, and hypotension, resolved, and he was discharged on day 5. This case demonstrates that tocilizumab can be an effective intervention in managing severe HSRs associated with CRRs. To our knowledge, this is the first reported instance of tocilizumab successfully salvaging a patient experiencing oxaliplatin-induced HSR. Nevertheless, further research is required to validate the efficacy of tocilizumab in treating oxaliplatin-induced HSRs.

## 1 Introduction

Oxaliplatin, a third-generation platinum-based cytotoxic agent, is widely used in the treatment of various malignant tumors, including lymphoma. HSRs to oxaliplatin are not uncommon and can, in some cases, be severe. The reported incidence of HSRs to oxaliplatin ranges from 1% to 25% (Pagani et al., 2022). Severe reactions can complicate ongoing treatment, reduce therapeutic efficacy, and, in extreme cases, pose a life-threatening risk to patients. Therefore, effective management of HSRs is essential. Standard management strategies typically include desensitization protocols, antihistamine therapy, and symptomatic treatments. However, these conventional approaches may prove ineffective in patients with substantial IL-6 elevation and severe symptoms such as fever, chest pain, and hypotension (Silver et al., 2020). In such cases, IL-6 receptor antagonists may offer a promising therapeutic option. Tocilizumab, an IL-6 receptor antagonist, has been successfully employed in the management of cytokine release syndrome (CRS) associated with CAR-T cell therapy (Tvedt et al., 2021). Here, we report what we believe to be the first documented case in which tocilizumab provided rapid symptomatic relief in a patient experiencing severe HSR following oxaliplatin treatment.

## 2 Case presentation

A 65-year-old male presented to the hospital with a 3-month history of nasal congestion and masses on the left side of his face, left upper abdomen, and left thigh for the past month. The patient has no notable medical history, smoking or alcohol use, or family history. There have been no recent vaccinations or infections. Nasal endoscopy revealed a neoplasm in the nasal cavity, and pathological examination confirmed the diagnosis of extranodal NK/T-cell lymphoma. A positron emission tomography-computed tomography (PET-CT) scan demonstrated tumor involvement in both nasal cavities, both ethmoid sinuses, the upper palate, the posterior parietal wall of the nasopharynx, both cervical lymph nodes, the right adrenal gland, the left hypochondriac region, and the skin of the left thigh. Based on these findings, the patient was diagnosed with stage IV extranodal NK/T-cell lymphoma in March 2022. Serum EBV-DNA level was 4,660 copies/mL. Bone biopsy results were negative, and routine laboratory examinations were within normal limits. The patient had no history of allergies or any known factors associated with HSRs. In April 2022, the patient began a regimen of immunochemotherapy with the PP-Gemox protocol, consisting of pegaspargase 3750 IU on day 1, gemcitabine 1800 mg on day 1, oxaliplatin 170 mg on day 1, and toripalimab 240 mg on day 0, administered every 3 weeks. Toripalimab, an anti-PD-1 inhibitor, is manufactured by Shanghai Junshi Biosciences, China. After two cycles of treatment, significant clinical improvement was observed, including resolution of nasal congestion and reduction of the masses in the left side of his face, left upper abdomen, and left thigh. Serum EBV-DNA levels became undetectable, and PET-CT scans revealed only residual lesions in the nasal cavities and left submaxillary lymph nodes. The overall treatment response was classified as a large partial remission.

On 23 July 2022, the patient’s performance status was assessed as 0, with no symptoms and normal laboratory results. The physical examination revealed no significant abnormalities. Consequently, the fourth cycle of treatment was initiated. On day 0, toripalimab was administered, followed by the successful infusion of gemcitabine on day 1. However, approximately 50 mg into the oxaliplatin infusion on day 1, the patient developed chills, dyspnea, chest pain, and a fever of 38.2°C. Additional symptoms included tachycardia (heart rate 129 beats/min), tachypnea (respiratory rate 30 breaths/min), and hypoxemia, with blood oxygen saturation (SpO2) dropping to 84%. The physical examination revealed bronchial breath sounds in both lungs, with no audible dry or wet rales. The heart rate was rapid and regular. A hypersensitivity reaction to oxaliplatin was initially suspected, with other potential conditions, including pulmonary embolism (PE), infection, and myocardial infarction, also considered. Immediate interventions included intramuscular promethazine (25 mg), intravenous dexamethasone (10 mg), and oxygen therapy (10 L/min via mask). Despite these measures, the patient’s temperature rose to over 39°C, and blood pressure dropped to 70-80/50-40 mmHg. Blood gas analysis revealed: PaO2 57.9 mmHg, PaCO2 32.8 mmHg, pH 7.392, HCO3- 19.5 mmol/L, BE -4.6 mmol/L, and lactic acid 4.18 mmol/L. Laboratory results showed elevated procalcitonin (1.95 ng/mL), IL-6 (>5,000 pg/mL), D-dimer (9.95 mg/L FEU), and fibrin/fibrinogen degradation products (23 mg/L). The white blood cell count was 1.72 × 10^9/L, and hemoglobin was 117 g/L. Platelet count was 139 × 10^9/L. Hepatic and renal functions were within normal limits, with the exception of mild hypokalemia (3.26 mmol/L). The electrocardiogram (ECG) revealed sinus tachycardia and secondary ST-T changes. Echocardiography showed no significant abnormalities. Myoglobin levels were 90.13 ng/mL, while troponin and brain natriuretic peptide levels were within normal limits. No notable abnormalities were observed in dynamic ECG monitoring or myocardial biomarkers. Myocardial infarction was excluded. In chest CT, scattered inflammation was observed in both lungs, particularly in the lower lobes, accompanied by a small amount of pleural effusion in both sides of the chest. Blood culture results were negative. Computer tomography pulmonary angiography ruled out the possibility of PE. Based on the clinical presentation, including sudden chills, dyspnea, and chest pain following oxaliplatin infusion, as well as physical examination findings of increased breath sounds in both lungs and results of chest CT and blood culture, we suspect an allergic reaction to oxaliplatin rather than infection. The patient was considered with a grade 4 hypersensitivity reaction (HSR) to oxaliplatin, as per the Common Terminology Criteria for Adverse Events (CTCAE v5.0). As a result, a series of interventions were initiated, including intramuscular epinephrine (0.5 mg) and intravenous dopamine (200 mg in 30 mL 0.9% NaCl, administered at 5 mL/h via micropump) to support blood pressure, along with continuous positive airway pressure to enhance oxygenation.

**FIGURE 1:**
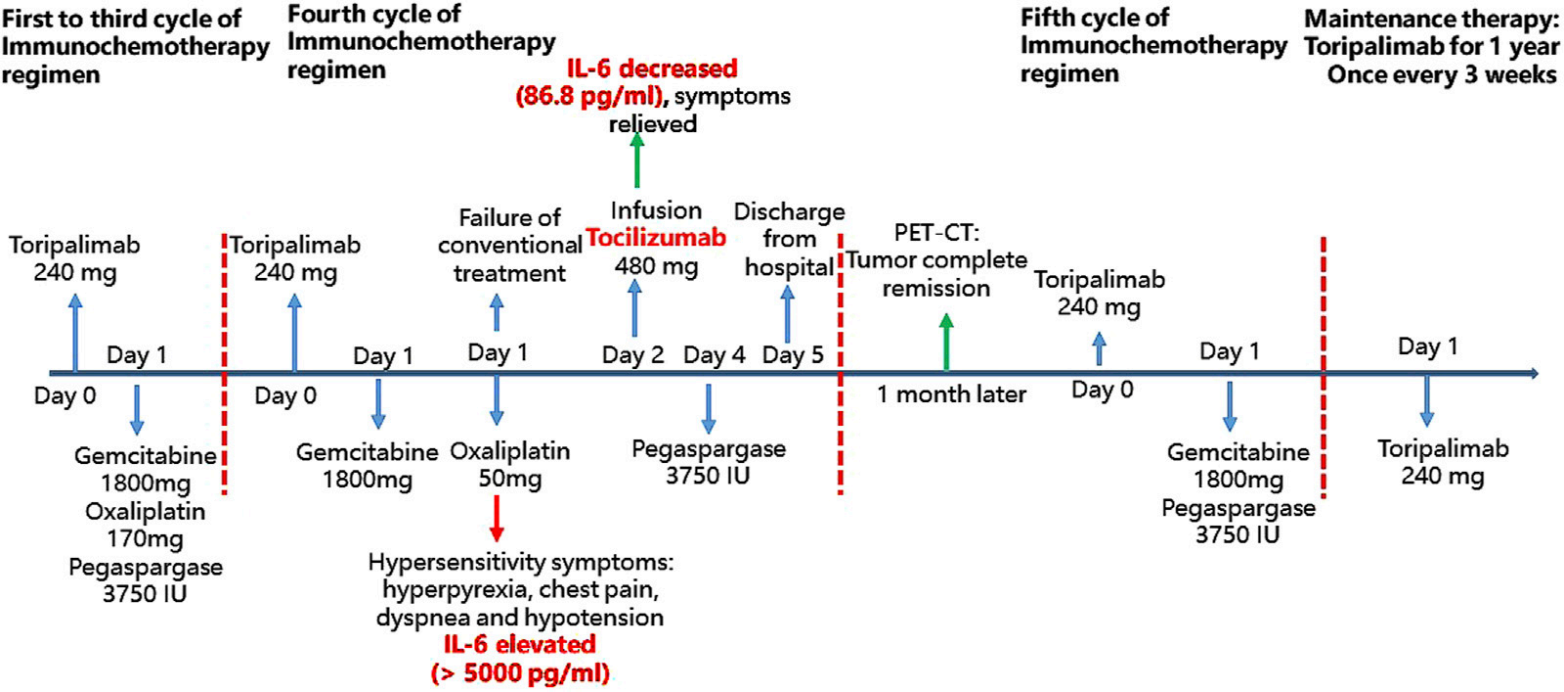
The patient’s entire treatment and monitoring process from oxaliplatin exposure to post-treatment with tocilizumab. In the first three cycles of immunochemotherapy, toripalimab 240 mg was infused on day 0, followed by gemcitabine 1800 mg, oxaliplatin 170 mg, and pegaspargase 3750 IU on day 1. During the fourth cycle, toripalimab was administered on day 0, and gemcitabine was infused successfully on day 1. However, after approximately 50 mg of oxaliplatin, the patient developed hypersensitivity symptoms and elevated IL-6 levels (>5,000 pg/mL). Conventional treatments failed to alleviate symptoms, including fever and dyspnea. On day 2, following tocilizumab infusion, IL-6 levels decreased to 86.8 pg/mL, and the patient’s symptoms improved. On day 4, pegaspargase 3750 IU was administered, and the patient was discharged on day 5. One month later, PET-CT showed complete tumor remission. In the fifth cycle, the patient received toripalimab, gemcitabine, and pegaspargase, followed by toripalimab for maintenance therapy for 1 year.

Despite approximately 8 h of the aforementioned treatments, the patient’s shivering subsided; however, hyperpyrexia (38.8°C–39°C), chest pain, dyspnea, and hypotension persisted. Considering that the HSR to oxaliplatin was associated with a cytokine release reaction and a significant elevation in IL-6 levels, tocilizumab (480 mg, 8 mg/kg) was administered on 25 July 2022. Several hours following the infusion of tocilizumab, the patient’s temperature returned to normal, and both dyspnea and hypotension resolved. Furthermore, IL-6 levels decreased markedly to 86.8 pg/mL. The patient’s condition improved markedly following the administration of tocilizumab, which further corroborated our diagnostic hypothesis. There were no additional adverse reactions observed over the subsequent days. On 27 July 2022, the patient received 3750 IU of pegaspargase and was discharged on 28 July 2022. No further anti-tumor therapies were administered post-discharge. In August 2022, serum EBV-DNA levels were undetectable, and PET-CT imaging revealed complete resolution of tumor metabolism, indicating a state of complete remission. The patient’s hypersensitivity to oxaliplatin led to the decision to exclude this agent and proceed with the fifth cycle, continuing with the remaining three chemotherapy drugs. After completing five cycles of chemotherapy, the patient transitioned to maintenance therapy with single-agent toripalimab for 1 year. The patient remains alive and free of recurrence. The patient’s entire treatment and monitoring process from exposure to oxaliplatin to post-treatment with tocilizumab is shown in Figure 1.

## 3 Discussion

HSRs are abnormal adaptive immune responses resulting from physiological dysfunction or tissue damage triggered by specific antigens. HSRs to chemotherapy are common with platinum salts (5%–46%), taxanes (4%–10%), and L-asparaginase (6%–43%) (ALMuhizi et al., 2022). The primary systems for grading HSR severity are the Common Terminology Criteria for Adverse Events (CTCAE, Table 1) and Brown’s grading system. The CTCAE focuses on infusion-related chemotherapy events, while Brown’s system addresses allergic reactions to various drugs (National Cancer Institute, 2017; Brown, 2004). Treatment for HSRs is critical. For grade 1 (CTCAE v5.0) or mild reactions (Brown), stopping the oxaliplatin infusion and replacing it with saline can relieve symptoms. For grade 2 or 3 (CTCAE v5.0) or moderate reactions (Brown), oxygen supplementation and appropriate medications are required. Antihistamines can alleviate itching, urticaria, and angioedema. Arachidonic acid pathway inhibitors (prostaglandin and leukotriene inhibitors) help mitigate gastrointestinal and respiratory symptoms. Corticosteroids are effective for delayed reactions and cytokine release reactions. Opioids like meperidine help relieve chills and rigors. In severe anaphylaxis (grade 4 or life-threatening), intramuscular epinephrine (0.1%, 0.01 mg/kg) is the primary treatment, with vasoactive drugs and ventilator support if necessary (ALMuhizi et al., 2022; Miyamoto et al., 2015). Rechallenge may be attempted after mild or moderate HSRs, but it carries a high risk after severe reactions.

**TABLE 1:** CTCAE v5.0 for infusion-related reactions (National Cancer Institute, 2017).

Grade	Severity and definitions
Grade 1	Mild; asymptomatic or mild symptoms; clinical or diagnostic observations only; intervention not indicated
Grade 2	Moderate; therapy or infusion interruption indicated but responds promptly to symptomatic treatment (e.g., antihistamines, corticosteroids, opioids, intravenous fluids)
Grade 3	Severe or medically significant but not immediately life-threatening; hospitalization or prolongation of hospitalization indicated; disabling; limiting self-care activity of daily living
Grade 4	Life-threatening consequences; urgent intervention indicated
Grade 5	Death related to adverse event

HSRs to oxaliplatin occur in 1%–25% of patients, with severe reactions occurring in less than 1%. These reactions can arise at any point during treatment but are most commonly observed during the sixth cycle. Key risk factors for HSRs to platinum-based drugs include prior exposure to the drug, a history of HSRs to other medications, increased severity of previous reactions, younger age (under 60 years), female gender, and more severe prior reactions (Pagani et al., 2022; Tham et al., 2015).

Routine treatments for oxaliplatin-induced HSRs are similar to those for other chemotherapeutic agents. However, these strategies may be ineffective in some patients. A study suggests that HSRs to oxaliplatin, characterized by elevated levels of TNF-α, IL-6, and other cytokines, are classified as CRRs. In these cases, an IL-6 receptor antagonist may offer an effective treatment option (Silver et al., 2020; Miyamoto et al., 2015). In CRRs with significantly elevated IL-6 (up to a 40-fold increase), IL-6 plays a central role in the resulting inflammatory storm of severe hypersensitivity reactions, autoimmune diseases, and infections like COVID-19 (Nepal and Gazeley, 2023; Pelaia et al., 2021; Scott et al., 2023). IL-6 is an inflammatory cytokine and low-molecular-weight glycoprotein secreted by lymphocytes (T cells and B cells) and non-lymphocytes (monocytes, dendritic cells, and tumor cells). IL-6 is a pleiotropic cytokine that exerts a wide array of immunomodulatory effects (Nepal and Gazeley, 2023; Mihara et al., 2012; Crayne et al., 2019; Romano et al., 1997; Hashizume et al., 2009). Its signaling is mediated through three distinct molecular pathways. IL-6 can bind to the membrane-bound IL-6R to initiate classic cis-signaling, or it can bind to soluble IL-6R to activate trans-signaling. Additionally, IL-6 can be presented on dendritic cells via their surface-bound IL-6R to activate T lymphocytes, a mechanism referred to as trans-presentation. Classic cis-signaling plays a central role in regulating both adaptive and innate immune responses, influencing the activation and function of critical immune cells such as T and B cells, natural killer cells, neutrophils, and macrophages. This pathway is particularly involved in the initiation and amplification of cytokine storms. IL-6 trans-signaling, on the other hand, is crucial in driving the development of systemic cytokine storms, as it promotes the synthesis of additional IL-6 and other pro-inflammatory mediators, including vascular endothelial growth factor (VEGF). It also increases the expression of E-cadherin, a cell adhesion molecule. Both VEGF and E-cadherin contribute to enhanced vascular permeability and endothelial leakage, key features of lung injury (Chen et al., 2021). Moreover, IL-6 trans-presentation supports the differentiation of T cells into a highly destructive phenotype, amplifying immune-mediated tissue damage (Garbers et al., 2018; Heink et al., 2017). Ultimately, the mechanisms of cis-signaling, trans-signaling, and trans-presentation converge on common intracellular signaling pathways, primarily involving the dimerization of gp130 and activation of key cascades such as JAK/STAT, MAPK, and PI3K. These pathways facilitate the transduction of IL-6 receptor-mediated signals to the nucleus, where they promote the expression of genes associated with cellular growth and proliferation, influencing a variety of cellular responses (Pelaia et al., 2021).

Tocilizumab, an IL-6 receptor antagonist and monoclonal antibody, can block all three forms of IL-6 signaling. Tocilizumab is indicated for conditions such as CRS, rheumatoid arthritis, systemic juvenile idiopathic arthritis, polyarticular juvenile idiopathic arthritis, giant cell arteritis, systemic sclerosis-related interstitial lung disease and COVID-19 (Nepal and Gazeley, 2023; Pelaia et al., 2021; Perl et al., 2024). In these situations, elevated levels of circulating cytokines, including IL-6, are common. In our case, the patient presented with symptoms consistent with a CRR, including fever (>38°C), chest pain, a marked rise in IL-6, dyspnea, and hypotension. These clinical features strongly suggested that the patient’s hypersensitivity reaction HSR was likely a CRR. Based on relevant studies, we administered tocilizumab, leading to a rapid resolution of symptoms as IL-6 levels decreased (Tvedt et al., 2021; Lee et al., 2014; Jimenez-Rodriguez et al., 2024). We hypothesize that the patient’s HSR was CRR-like, given the symptomatology and elevated IL-6 levels. Although tocilizumab proved effective in this case, further research is required to refine the diagnostic criteria for CRR-like HSRs and to better understand the efficacy and underlying mechanisms of tocilizumab in treating oxaliplatin-induced HSRs.

## 4 Conclusion

HSRs to oxaliplatin are common and can present with diverse manifestations, affecting various organs and systems due to different underlying mechanisms. While routine treatments are effective for many HSRs, conventional strategies may prove inadequate in cases of CRRs. Our case underscores that when CRR is suspected—based on symptoms such as fever, chills, dyspnea and notably elevated IL-6 levels—an IL-6 receptor antagonist, such as tocilizumab, should be considered. Further studies are needed to fully assess the efficacy of tocilizumab in managing oxaliplatin-induced HSRs.

## Data Availability

The original contributions presented in the study are included in the article/supplementary material, further inquiries can be directed to the corresponding author/s.

## Abbreviations

HSR, hypersensitivity reaction; CRR, cytokine release reaction; IL-6, interleukin-6; IL-6R, IL-6 receptor; CRS, cytokine release syndrome; PET-CT, positron emission computed tomography; PE, pulmonary embolism; ECG, electrocardiogram; CTCAE, Common Terminology Criteria for Adverse Events; VEGF, vascular endothelial growth factor.
